# Book Review

**Published:** 1902-06

**Authors:** 


**William Hunter.** By R. Hingston Fox. Pp. viii., 75. London: H. K. Lewis. 1901.—We have read this short story of the life of William Hunter, anatomist, physician, and obstetrician, with much pleasure. The author's literary style, which is scholarly and elegant, as well as the subject matter, make the book a most readable one. In addition to the account of the life and work of William Hunter, interesting notices are given of the lives of Cullen, Smellie, Fothergill, and Baillie, all friends of Hunter, and portraits of Hunter and of these famous contemporaries are introduced. It is apparently impossible to discover how much of the credit for the discovery of the mode of circulation of the maternal and fœtal blood in the placenta should be attributed to William and how much to John Hunter. Much of the work which led to the discovery was done by the two brothers in conjunction, and later each claimed the chief credit. The crowning work of William Hunter was, however, his demonstration of the "Anatomy of the Human Gravid Uterus," culminating in the publication of the book bearing that title. The author quotes Matthews Duncan's statement that this "immortal work is one of the stable foundations of the science and art of midwifery and cannot fail, in all future ages, to be as valuable and useful as it now is."

**On the Cure of the Morphia Habit without Suffering (Physiological Demorphinisation).** By Oscar Jennings, M.D. Second Edition. Pp. xii., 211. London: Baillière, Tindall and Cox. 1901.—For more than ten years the author of this book, having first of all cured himself of this distressing condition, has been the means of curing many others, and has been quite the recognised authority thereon. The means of treatment advocated are: heart tonics, bicarbonate of soda, and hot-air baths—a therapeutic triad, together with the special mode of reduction of doses by means of rectal injections. Numerous interesting cases are recorded, and these show the way in which the details of treatment may be carried on in spite of many difficulties.

**Surgical Applied Anatomy.** By Sir FREDERICK TREVES, K.C.V.O., C.B. New [Fourth] Edition. Revised by the Author, with the assistance of ARTHUR KEITH, M.D. Pp. xii., 571. London: Cassell and Company, Limited. 1891.—This popular work has reached its twenty-fourth thousand, and is now so well known by students and practitioners as to need no recommendation from us. It is full of useful information given in a pleasant form. It is modelled somewhat after the style of Hilton's *Rest and Pain*, which it resembles in presenting a number of important anatomical details illustrated with interesting surgical embellishments. We know of no other book on surgical anatomy which is so eminently readable as this.

**A Text-book of Embryology.** By J. C. HEISLER, M.D. Pp. 405. Philadelphia and London: W. B. Saunders. 1899.—This treatise gives a fairly full account of the development of man, illustrated by the usual figures. The feature which distinguishes it is an elaborate table showing the size of the embryo and the state of development of the various organs at various ages. It is a most useful compilation and deserves to be widely known.

**The Essentials of Practical Bacteriology.** By H. J. CURTIS, M.D. Pp. xvi., 291. London: Longmans, Green and Co. 1900.—This work is eminently suited for a laboratory text-book for students of bacteriology, and we know of no other which so fully serves this purpose. The directions with regard to technique are clear and concise, and the particular study of each organism is considered in a separate lesson. Tables are supplied in the appendix, which will prove of great service to the beginner in helping to differentiate between the commoner varieties of organisms. The subjects of cancer and ringworm are considered at some length, and the more recent work in connection with these two diseases is given in detail.

**Principles of Surgery.** By N. SENN, M.D., LL.D. Third Edition. Pp. xiv., 699. Philadelphia and Chicago: F. A. Davis Company. 1901.—A thorough revision of this valuable work has been made, and in its present form it ranks in the forefront of scientific surgery. Amongst the new matter is a welcome chapter on blastomycetic dermatitis—a comparatively new disease which has hitherto been mistaken for one of the phases of tuberculosis. In reading the clear and didactic statements of the author, one is tempted to wish that such solid principles, founded on fact and experiment, could be substituted for some of the ephemeral details of treatment with which the modern student is too laboriously crammed. We can heartily recommend the book to those who desire an original, scientific, and intelligible exposition of the principles of surgery.

**When to Operate in Appendicitis.** By C. MANSELL MOULLIN, M.D. Second Edition. Pp. 39. London: John Bale, Sons, and Danielsson, Ltd. 1901.—These clinical lectures—for such they are—were originally published in the *Clinical Journal.* The author, like our American *confrères*, appears to favour operation in acute inflammations which are not showing any sign of improvement in twelve or twenty-four hours. To him the "question is a very simple one," and he asks—equally simply—which of the two offers the best prospect for the patient, an early operation or "leaving things to chance"? He has apparently forgotten the middle course of bringing an intelligent watchfulness to bear on the progress of the case when we decide not to operate within twenty-four hours! The book is, however, lucidly written, and should certainly be in the hands of all practitioners who have been in the habit of "leaving things to chance."

**Tumours, Innocent and Malignant.** By J. BLAND-SUTTON. New Edition. Pp. 566. Cassell and Company, Limited. 1901.—The former edition ranks as a classic; and the present edition, with its additions and corrections, is not altered in character from the previous issue. The whole work is eminently scientific, original, and concise, and bears witness on every page to the painstaking efforts of the author, who has endeavoured to put the classification of tumours on an intelligible basis. The whole treatment of tumours is tersely summed up in the preface as consisting in their removal, wherever this is practicable at the earliest possible moment; but it is surely an anachronism to suggest that in the treatment of cancer of the breast "many surgeons" are in the habit of "rudely pulling out the axillary lymph-glands one by one" (p. 275). The book should be possessed by all who are interested in the evolution of tumours.

**Tumours of the Urinary Bladder.** By E. HURRY FENWICK. Pp. viii., 115. London: J. & A. Churchill. 1901.—We think the author might have presented the results of his experience in a more readable form and with more method in arrangement than here given, and might have added an index in place of the list of cases of which "the scant details do not, I regret to say, convey any knowledge to the reader." The rules of technique are worth repeating: (1) Always cystoscope, never sound for symptomless hæmaturia; (2) A gentle rectal examination should be the first step in the inquiry; (3) Dark hæmaturias without clots rarely need any preparatory washing out for clear cystoscopy—a diuretic often suffices; (4) Always cystoscope and operate, if necessary, at the same sitting if the bladder has to be washed out. The author calls attention to some of the difficulties and to the dangers of cystoscopy, which should be fully understood before anyone attempts the procedure. His classification of bladder tumours into (*a*) the benign papilloma, (*b*) the papillomatous carcinoma, and (*c*) the bald carcinoma, is borne out in the tables to be a clinically convenient one. We note that Mr. Fenwick no longer uses syphon-drainage after his supra-pubic operations. Many useful hints will be found throughout the book.

**Gonorrhœal Arthritis: its Pathology, Symptoms, and Treatment.** By L. VERNON JONES, M.D. Pp. 52. London: H. K. Lewis. 1901.—The account of gonorrhœal arthritis given in this little book is not much fuller than that in most of the ordinary text-books of medicine and surgery. Only the second half of the book deals strictly with arthritis. In the first half the bacteriology of gonorrhœa and the phenomena of inflammation in general are considered. For the support of the inflamed joints the author greatly prefers leather splints to any others. No mention is made of the possibility of increasing the after-stiffness in the joints by keeping splints applied for too long a time; the cautions given are all against leaving off the splints too soon. We think the hot-air and electric bath treatment deserved mention. The book, though it contains nothing original or new, may be of use to practitioners who have not access to the larger works on medicine and surgery, though we think that all practitioners should consider it a duty to get access to good medical literature.

**Tubercular Disease of the Hip-Joint.** By J. GRANT ANDREW, M.B. Pp. 69. Glasgow: Alex. MacDougall. 1901.—This is a reprint of a paper read before the Glasgow Medico-Chirurgical Society in favour of the establishment, for Glasgow, of a similar special hospital to the Alexandra institution for London. It is a valuable statistical record to show that only long hospital treatment (five months on the average at the Alexandra), with prolonged observation afterwards, can give to the poor the same results as can be obtained for the rich. In general hospitals, from various reasons—largely the demand upon the beds—time-saving excision becomes a necessity. In private, and under special hospital conditions, Nature can be left to eliminate the diseased focus if adequate time be allowed. It is the prolonged rest in the early stage that is so important and so difficult to provide for in a general hospital.

**Transactions of the American Surgical Association.** Vol. xviii. Edited by DE FOREST WILLARD, M.D., Ph.D. Philadelphia: William J. Dornan. 1900.—We have grown accustomed to regard this volume as one of the most important of the yearly contributions to surgical literature, and the present one serves to confirm that view. It contains the papers read before the Association at the meeting held in May, 1900.

More than half the book is taken up with a series of articles on gastric surgery, written by various leading American surgeons, and these articles are of such excellence that no one interested in that field of surgery can afford to miss reading them. The remaining articles in the book on sundry other surgical topics are of almost equal merit.

**Syphilis and other Venereal Diseases.** By H. DE MÉRIC. Pp. vii., 132. London: Baillière, Tindall and Cox. 1901.—The writer explains why he has thought it well to exclude the moral or religious side of the question in writing this book. We hardly think such an explanation needful, for surely such topics would be entirely out of place in any medical book. The book contains a short account of venereal diseases, in which the author's own experience is freely given, and it is written in an interesting way. His experience seems to have been chiefly gained in the French hospital to which he is surgeon. The work can hardly be considered a standard one for reference. We fear the method of puncturing an inflamed testicle in the out-patient room, although it may give temporary relief, may, without thorough antiseptic precautions, lead to suppuration in the testicle, and no mention is made of such precautions.

**Studies from the Department of Pathology of the College of Physicians and Surgeons, Columbia University, N.Y.** Vol. VII. For the Collegiate Year, 1899—1900. Reprints.—This volume contains many valuable papers. One on the antitoxin treatment of tetanus, by Dr. Moschcowitz, is very comprehensive. He has collected and abstracted no fewer than 338 cases: 196 recovered, 142 died, a mortality of 42.01 per cent.; but, as the editor remarks in a footnote, this must not be taken as a reliable estimate of the mortality of cases treated by antitoxin, since more successful than unsuccessful cases are reported. As a comment upon this, it is worthy of note that all the recorded cases of tetanus treated in Italy with Tizzoni's antitoxin recovered. Another interesting paper is upon poisonous snakes and snake-bite, by Gustav Langmann. A statement as to the fatality of the bite of the European viper is somewhat surprising. In Loire Inférieure sixty-two deaths are said to have occurred in six years from the bite of the common viper. The value of antivenine as a remedy is discussed, and in spite of contradictory reports it is considered that it possesses some curative power; but the opportunities for using it successfully are of necessity rare. A point of interest, bearing perhaps not only upon the treatment of snake-bite but suggestive as to the value of drugs in general medicine, is the statement that cholesterin extracted from biliary calculi and from carrots, and tyrosin separated from the bulb of the dahlia and from mushrooms, appear to have the power of immunizing against viper venom. It is also mentioned that in America the Indians have used a plant, miscania gaucho, with reputed success in the treatment of snake-bite.

There are other valuable papers, amongst which may be mentioned traumatic hemorrhages into the spinal cord, by Dr. Pearce Bailey. Hemorrhage into the spinal canal is spoken of as rare, and hemorrhages into the spinal cord as more common than is generally supposed. Such hemorrhages are usually focal, but in rare instances may be disseminated. The grey matter of the cervical region is the usual seat of a focal hemorrhage. It follows that paralysis of one or of both arms commonly occurs, and with this may be associated loss of sensation to temperature and pain. If the hemorrhage be large, the legs may also suffer. The prognosis is not necessarily bad. Should the hemorrhage have been sufficient to destroy cells of the anterior horns, wasting of the hands or arms will follow, but the paralysis in the legs generally disappears.

**A Text-book of Practical Obstetrics.** By Egbert H. Grandin, M.D., with the collaboration of George W. Jarman, M.D. Third Edition. Pp. xv., 511. Philadelphia: F. A. Davis Company. 1900.—This work is quite properly described by its title; it is a very thorough and reliable guide to the methods of dealing practically with the various situations which arise in midwifery. We can especially commend the section on the pathology of pregnancy, which deals with the subject in great detail. We note with surprise, however, that the authors do not call the attention of the reader, when dealing with the nephritis of pregnancy, to the importance of not only testing for urea—by which, we presume, they mean its volumetric estimation—but also of keeping a daily record of the amount excreted as well as the percentage found, since this affords a most important guide to the condition of the excretory power of the kidneys. We note with appreciation the advice of the authors as to saline transfusion, and also their remarks as to the various methods of its application and their respective value. There can be no doubt in the minds of any who have made use of the methods described, that their failure is in most cases due to the fact that they have not been pushed by frequent repetition. In dealing with puerperal sepsis, the authors indicate very clearly, in our opinion, the correct attitude towards the extirpation of the pelvic organs for the relief of septic fever: this operation one would expect to be rarely justifiable. The book is heavily illustrated, most of the plates being taken from photographs. We must confess that in many cases we fail to see the advantage of the plates from a practical point of view; occasionally we come across one which, being a photograph, shows up some fault in technique, *e.g.*, a gentleman with aseptic hands is engaged in a manipulation and wearing a ring!! One plate, showing the application of the binder, rather indicates the apparatus being loose where it should be tight, and *vice versa.* Many of the plates, especially photographs of internal manipulations with the internal hand necessarily omitted, appear to us quite superfluous: we fail to see what knowledge can be gained from them. Taking the work as a whole we consider it a sound and sensible exposition of the essential points in the treatment of either a normal or complicated case.

**The Journal of Obstetrics and Gynæcology of the British Empire.** Vol. I., No. I. January, 1902. Edited by ALBAN H. G. DORAN. London: Baillière, Tindall and Cox.—The aim of the promoters of this new journal, as declared in the preface of this its first number, is to make it "a complete and impartial record of British work, an exponent of British obstetrical and gynæcological practice, and a summary of contemporary thought and achievement in obstetrics and gynæcology throughout the world." With these high aims we are naturally in entire sympathy. We like the title of the journal. The cover bears a long list of obstetricians and gynæcologists, all British in the wider sense, who have agreed to help in the work of the journal. Issued under the control of such a large number of distinguished specialists, its success seems assured. The specimen copy to hand begins well. The first eighty-eight pages contain five original articles and one so-called critical essay. The five original papers, though interesting, are not of extraordinary merit, and we found the essay more instructive than any of them. Thirty-two pages are devoted to abstracts from current literature, and the abstracts have been very well written. Finally, nine pages are given to reviews of books and reports of societies. We should like to see the table of contents not split into two parts on separate pages, and not preceded by and mixed up with advertisements, which tend to obscure it. We think, also, that it would be a distinct advantage to have each abstract from current literature individually noticed in the table of contents in some such way as they are in the German *Centralblatts*, or even more systematically than in these. The binding, too, leaves something to be desired. Our own copy showed signs of disintegration before we had finished reading it. We hope the day will come when this journal will be known far and wide, in the empire and abroad, as one of the very highest order. We venture to hope that its success may be such that the physicians and the surgeons of the Empire will follow the example of the obstetricians and gynæcologists, and combine together to issue two more journals of the British Empire, devoted respectively to medical and surgical science, and that the journal under review, or the hypothetical three, may serve to encourage and stimulate the profession throughout the land to make greater efforts for the advancement of the medical sciences, and that aided by these publications Greater Britain may in the end deserve and gain fame as great in the medical field as she has already done in other spheres of labour.

